# Impulse oscillometry for detection of small airway dysfunction in subjects with chronic respiratory symptoms and preserved pulmonary function

**DOI:** 10.1186/s12931-021-01662-7

**Published:** 2021-02-24

**Authors:** Liang-Yuan Li, Tian-Sheng Yan, Jing Yang, Yu-Qi Li, Lin-Xi Fu, Lan Lan, Bin-Miao Liang, Mao-Yun Wang, Feng-Ming Luo

**Affiliations:** 1 Department of Respiratory and Critical Care Medicine, West China School of Medicine and West China Hospital, Sichuan University, No. 37 Guoxue Alley, Chengdu, 610041 China; 2 Department of Critical Care Medicine, West China School of Medicine and West China Hospital, Sichuan University, Chengdu, 610041 China

**Keywords:** Small airway dysfunction, Spirometry, Impulse oscillometry, Preserved pulmonary function

## Abstract

**Background:**

Subjects with chronic respiratory symptoms and preserved pulmonary function (PPF) may have small airway dysfunction (SAD). As the most common means to detect SAD, spirometry needs good cooperation and its reliability is controversial. Impulse oscillometry (IOS) may complete the deficiency of spirometry and have higher sensitivity. We aimed to explore the diagnostic value of IOS to detect SAD in symptomatic subjects with PPF.

**Methods:**

The evaluation of symptoms, spirometry and IOS results in 209 subjects with chronic respiratory symptoms and PPF were assessed. ROC curves of IOS to detect SAD were analyzed.

**Results:**

209 subjects with chronic respiratory symptoms and PPF were included. Subjects who reported sputum had higher R5–R20 and Fres than those who didn’t. Subjects with dyspnea had higher R5, R5–R20 and AX than those without. CAT and mMRC scores correlated better with IOS parameters than with spirometry. R5, R5–R20, AX and Fres in subjects with SAD (n = 42) significantly increased compared to those without. Cutoff values for IOS parameters to detect SAD were 0.30 kPa/L s for R5, 0.015 kPa/L s for R5–R20, 0.30 kPa/L for AX and 11.23 Hz for Fres. Fres has the largest AUC (0.665, P = 0.001) among these parameters. Compared with spirometry, prevalence of SAD was higher when measured with IOS. R5 could detect the most SAD subjects with a prevalence of 60.77% and a sensitivity of 81% (AUC = 0.659, P = 0.002).

**Conclusion:**

IOS is more sensitive to detect SAD than spirometry in subjects with chronic respiratory symptoms and PPF, and it correlates better with symptoms. IOS could be an additional method for SAD detection in the early stage of diseases.

## Background

Chronic obstructive pulmonary disease (COPD) and asthma are common chronic respiratory diseases, which may involve small airways. Prospective evidence showed that small airway dysfunction (SAD) might occur prior to the development of COPD and asthma [1–3]. Cough, sputum, dyspnea and wheeze are common symptoms in COPD and asthma patients. SAD may exist in some subjects suffered from the above symptoms but with preserved pulmonary function (PPF, the forced expiratory volume in 1st second (FEV_1_)/forced vital capacity (FVC) ratio ≥ 0.70 [4]) and negative airway hyper-responsiveness (AHR) or bronchial reversibility (BR), i.e. not meeting the pulmonary function criteria of COPD or asthma. A large multistage stratified sampling survey showed that more than 40% of Chinese adults aged 20 or older have spirometry-defined SAD [5]. Since the heavy burden of SAD, it is of great importance to take efforts on its early detection and intervention.

Called as “quiet zone”, small airways (< 2 mm internal diameters) account for a small proportion of total airway resistance because of their large cross-sectional area. Currently, the most widely used method in clinic to assess small airway function is spirometry, and the adopted parameters are the forced expiratory flow between 25 and 75% of FVC (FEF_25–75%_), FEF at 50% of FVC (FEF_50%_) and FEF when 75% of FVC has been exhaled (FEF_75%_). However, the maneuver requires good cooperation of subjects, and the great variability of values makes their reliability not being unanimously recognized [6, 7].

With a different measuring principle from spirometry, impulse oscillometry (IOS) could measure the respiratory mechanical properties during quiet tidal breathing. Compared with spirometry, it is independent of the subjects’ efforts because of the externally superimposed oscillation signals [8]. Furthermore, it may have higher sensitivity in detecting SAD and seems to be better correlated with small airway structures [9–11]. Since IOS could reflect the viscous property of the respiratory system by resistance (Rrs), elastic and inertial property by reactance (Xrs), it might provide deeper information on individuals’ pathology changes when applied coupled with spirometry.

The present study aimed to explore the diagnostic value of IOS to detect SAD in subjects with chronic respiratory symptoms and PPF. We hypothesized that IOS could be a supplementary method to detect SAD, making up for the deficiency of spirometry, and improving the sensitivity of detection ability. We also intended to compare the correlation between symptoms and IOS as well as spirometry.

## Methods

### Study design and subject selection

This was a single-centered, observational study in which subjects were recruited and tested at the Pulmonary Function Laboratory of West China Hospital, Sichuan University, Chengdu, China between May 1st and September 1st, 2020.

To be included in this study, subjects had to be aged over 18 years and came to receive pulmonary function tests because of chronic respiratory symptoms. Besides, subjects were eligible when they fulfill the criteria of PPF (FEV_1_/FVC ≥ 0.70) [4]. Exclusion criteria were as follows: restrictive pulmonary diseases (FVC < 80% predicted), asthma, interstitial lung diseases, structural lung diseases covering active/previous tuberculosis and bronchiectasis, lung cancer, respiratory infection in 2 weeks, myocardial ischemia, history of pulmonary surgery, and incompletion of IOS because of incorrect tongue position, vocal cord closures or swallowing.

We also recruited never-smokers (≤ 1 pack-year of tobacco-smoking history) with normal chest radiogram and without current pulmonary diseases or unstable cardiovascular diseases as healthy controls.

Basic demographic variables including sex, age, weight, height, and body mass index (BMI) were collected. Subjects received IOS, spirometry, and completed a questionnaire covering qualitative and quantitative evaluation of symptoms. Also, bronchial provocation tests or bronchodilator tests were performed to exclude asthma.

The study was approved by the ethics committee of West China Hospital, Sichuan University, and all participants signed an informed consent before the procedure.

### Impulse oscillometry

The respiratory resistance and reactance were measured using IOS equipment (MS-IOS Jaeger) following protocols of ERS [8]. IOS was conducted before spirometry because forced expiration itself might change airway tone [12]. Pressure oscillations generated by a loudspeaker were superimposed onto normal tidal breathing through a mouthpiece for 30 to 45 s, which ranged from 5 to 35 Hz in frequency. Sitting upright, subjects were asked to wear a nasal clip and exert manual compression on their faces to minimize the influence of cheek vibration and air leak. Three trials were performed and mean values of the following parameters were recorded: respiratory resistance at 5 Hz (R5) and 20 Hz (R20), the difference between R5 and R20 (R5–R20), reactance at 5 Hz (X5), resonant frequency (Fres) and the area under reactance curve between Fres and 5 Hz (AX).

### Spirometry and bronchodilator/bronchial provocation test

Spirometry was performed by a full MasterScreen PFT System (Jaeger Corp. Germany) according to the American Thoracic Society (ATS)/European Respiratory Society (ERS) guidelines [13]. FEV_1_, FVC, FEV_1_/FVC, FEF_25–75%_, FEF_50%_ and FEF_75%_ were recorded as percentages of predicted values. The prediction equations are based on a large study of normal spirometry values in Chinese aged 4–80 years, which is recommended in the spirometry guideline in China [14]. Spirometry-SAD was defined as at least two of the three small airway indicators (FEF_25–75%_, FEF_50%_ and FEF_75%_) were less than 65% predicted value [5]. To exclude subjects with asthma, most subjects received bronchial provocation tests to confirm negative AHR, and the others received bronchodilator tests in the requirement of negative BR. Bronchial provocation tests were conducted with the Jaeger APS Pro system, following the recommendations of the ATS/ERS guideline [15]. Positive AHR was defined as the provocative dose inducing a 20% decrease of FEV_1_ (PD_20_FEV_1_) ≤ 2.5 mg. For subjects accepted bronchodilator tests, 400 μg salbutamol through a metered-dose inhaler was administered and spirometry was repeated after 15 min. BR was positive if the improvement of FEV_1_ or FVC or both is over 12% and 200 ml compared with baseline values before inhalation.

### Questionnaire

All participants accepted a questionnaire for qualitative and quantitative evaluation of chronic respiratory symptoms. Chronic respiratory symptoms included cough, sputum, wheeze and dyspnea [16]. Modified Medical Research Council dyspnea scale (mMRC) score and the COPD Assessment Test (CAT) were used to evaluate the severity of symptoms.

### Statistical analysis

Data analysis was performed with SPSS software version 26.0. Continuous variables were expressed as mean ± SD or median (interquartile range). Categorical variables were expressed as frequency and frequency percentages. Independent t (t’)-test, Chi-square test, and Mann–Whitney test were used for statistical difference inferences. Spearman correlation was chosen to determine relationships between spirometry, IOS parameters and symptom scores followed by Holm–Bonferroni correction. With at least two of the three small airway indicators of spirometry (FEF_25–75%_, FEF_50%_ and FEF_75%_) less than 65% predicted value as the standard of SAD, receiver operator characteristic curves (ROC) were conducted to evaluate the ability of IOS parameters to diagnose SAD. The area under the curve (AUC) and cutoff values were calculated. A P-value less than 0.05 was considered significant.

## Results

### Characteristics of study population and healthy controls

Demographics, baseline spirometry and IOS parameters of 85 healthy controls as well as 209 subjects with chronic respiratory symptoms and PPF are shown in Table 1. The quality of all performed spirometry tests were above Grade C according to the ATS/ERS guideline [13]. No significant differences were found in demographic characteristics between symptomatic subjects and healthy controls.

**Table 1:** Characteristics of healthy controls and subjects with chronic respiratory symptoms and PPF

	Symptomatic subjects with PPF (n = 209)	Healthy controls (n = 85)	P value
Demographics			
Age (years)	39.53 ± 12.79	36.92 ± 10.21	0.067
BMI (kg/m^2^)	22.09 ± 3.12	22.68 ± 2.57	0.123
Sex: male, n (%)	97 (46.4)	40 (47.1)	0.920
Spirometry			
FEV_1_ (% predicted)	104.61 ± 12.41	106.58 ± 10.73	0.201
FVC (% predicted)	105.90 ± 13.09	106.65 ± 12.85	0.656
FEV_1_/FVC	83.78 ± 6.97	85.16 ± 5.81	0.108
FEF_25–75%_ (% predicted)	86.48 ± 22.71	93.02 ± 21.01	0.023
FEF_50%_ (% predicted)	90.98 ± 22.95	100.53 ± 22.64	0.001
FEF_75%_ (% predicted)	81.37 ± 30.57	85.50 ± 27.61	0.281
IOS			
R5 (kPa/L s)	0.32 ± 0.07	0.28 ± 0.06	< 0.001
R20 (kPa/L s)	0.29 ± 0.06	0.26 ± 0.06	< 0.001
R5–R20 (kPa/L s)	0.0219 ± 0.0273	0.0193 ± 0.0238	0.449
X5 (kPa/L s)	− 0.1009 ± 0.0279	− 0.1007 ± 0.0280	0.966
Fres (Hz)	11.38 ± 2.58	10.49 ± 2.10	0.005
AX (kPa/L)	0.24 (0.17, 0.34)	0.20 (0.14, 0.30)	0.015

The data are presented as mean ± SD for normally distributed variables and median (interquartile range) for nonnormally distributed variables

*BMI* body mass index

As the best and most common indicators to judge the presence of airflow obstruction, FEV_1_ and FEV_1_/FVC were not significantly different in the two groups. Despite FEF_25–75%_ and FEF_50%_ of symptomatic subjects statistically lower than controls, they were still within the normal range.

For IOS parameters, R5, R20, Fres and AX were significantly higher in symptomatic subjects with PPF than healthy controls. But R5–R20 and X5 did not differ between groups.

### Relationship between symptoms, spirometry and IOS parameters

**Fig. 1:**
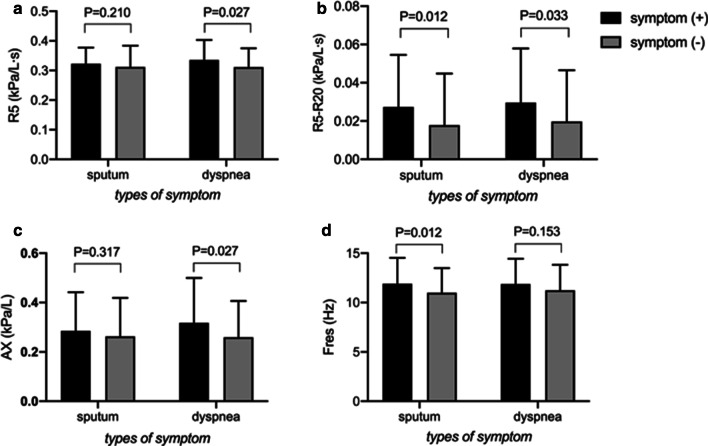
**a** R5, **b** R5–R20, **c** AX, **d** Fres were plotted for subjects with PPF from the symptom (+) versus symptom (−) groups according to the two specified symptoms (sputum and dyspnea). Bar charts represented mean + SD

In 209 subjects with chronic respiratory symptoms and PPF, no spirometry or IOS parameters were different between subjects with and without cough/wheeze. Subjects who reported symptom of sputum had higher R5–R20 (0.0274 ± 0.0271 kPa/L s versus 0.0179 ± 0.0269 kPa/L s, P = 0.012, Fig. 1b) and Fres (11.90 ± 2.62 Hz versus 11.00 ± 2.50 Hz, P = 0.012, Fig. 1d) compared those without sputum. Those who reported dyspnea had higher R5 (0.34 ± 0.07 kPa/L s versus 0.31 ± 0.06 kPa/L s, P = 0.027, Fig. 1a), R5–R20 (0.0296 ± 0.0284 kPa/L s versus 0.0198 ± 0.0268 kPa/L s, P = 0.033, Fig. 1b) and AX (0.32 ± 0.18 kPa/L versus 0.26 ± 0.15 kPa/L, P = 0.027, Fig. 1c) compared with those did not. Among spirometry parameters, only FEF_75%_ was significantly different between subjects with and without dyspnea (73.44 ± 28.78% predicted versus 83.54 ± 30.77% predicted, P = 0.049).

When it came to the quantitative evaluation of symptoms (Table 2), no spirometry parameters were associated with the mMRC score. However, R5, R5–R20, and AX were significantly correlated with the dyspnea score, although they did not survive conservative Bonferroni correction. As for the CAT score, significant correlations were observed in FEV_1_/FVC and FEF_75%_ for spirometry, but they failed to survive Bonferroni correction. All IOS parameters were significantly correlated with CAT score, especially R5, despite failures of R20 and R5–R20 to survive Bonferroni correction.

**Table 2:** Spearman correlation coefficients of symptom scores with spirometry and IOS parameters in subjects with chronic respiratory symptoms and PPF (n = 209)

Spirometry parameter	FEV_1_%	FVC %	FEV_1_ /FVC	FEF_25–75%_ %	FEF_50%_ %	FEF_75%_ %
mMRC	− 0.004	0.021	− 0.072	− 0.082	− 0.084	− 0.126
CAT	0.029	0.112	− 0.188**	− 0.135	− 0.124	− 0.169*

IOS parameter	R5	R20	R5–R20	X5	AX	Fres
mMRC	0.150*	0.111	0.162*	− 0.127	0.179**	0.131
CAT	0.243**	0.174*	0.150*	− 0.200**	0.204**	0.212**

FEV_1,_ FVC, FEF_25–75%_, FEF_50%_ and FEF_75%_ are expressed as % predicted

Values in italic indicate significant correlations after Bonferroni correction

*P < 0.05

**P < 0.01

FEV_1_/FVC, FEF_25–75%_, FEF_50%_ and FEF_75%_ were significantly correlated with all IOS parameters except for X5 after correlation analysis with Bonferroni correction (Table 3). The strongest correlation was observed between FEF_25–75%_, FEF_50%_, FEF_75%_ and R5 (r = 0.37, P < 0.001 for all). Moderate correlations were also found between R5–R20, AX, Fres and the three small airway parameters of spirometry.

**Table 3:** Correlation between spirometry and IOS parameters in subjects with chronic respiratory symptoms and PPF (n = 209)

IOS parameter	FEV_1_ (% predicted)		FVC (% predicted)		FEV_1_/FVC		FEF_25–75%_ (% predicted)		FEF_50%_ (% predicted)		FEF_75%_ (% predicted)	
	r	P value	r	P value	r	P value	r	P value	r	P value	r	P value
R5	− 0.15	0.033	0.04	0.526	− 0.23	0.001	− *0.37*	*< 0.001*	*−* *0.37*	*< 0.001*	*− 0.37*	*< 0.001*
R20	− 0.12	0.092	0.01	0.923	− 0.08	0.243	− *0.26*	*< 0.001*	*−* *0.28*	*< 0.001*	*− 0.23*	*0.001*
R5–R20	− 0.07	0.293	0.09	0.208	− *0.33*	< *0.001*	− *0.27*	*< 0.001*	*−* *0.23*	*0.001*	*− 0.35*	*< 0.001*
X5	0.13	0.068	0.09	0.211	− 0.02	0.791	0.19	0.005	0.19	0.005	0.16	0.018
AX	− 0.17	0.013	− 0.05	0.471	− 0.16	0.020	− *0.30*	*< 0.001*	− *0.28*	*< 0.001*	− *0.29*	*< 0.001*
Fres	− 0.13	0.069	0.03	0.623	− *0.28*	< *0.001*	− *0.30*	*< 0.001*	− *0.28*	*< 0.001*	− *0.31*	*< 0.001*

Values in italic indicate significant correlations after Bonferroni correction

### Characteristics of the subjects with and without SAD

Classified according to the presence of spirometry defined SAD, demographics and clinical features of the subjects with chronic respiratory symptoms and PPF were shown in Table 4. No differences were found in both qualitative and quantitative assessment of chronic respiratory symptoms. In spirometry parameters, not only the three small airway parameters (P < 0.001 for all) but also FEV_1_ and FEV_1_/FVC decreased in subjects with SAD.

**Table 4:** Features and spirometry parameters of subjects with and without spirometry-SAD

	SAD n = 42 (20.1%)	no SAD n = 167 (79.9%)	P value
Demographics and clinical history			
Age (years)	41.81 ± 12.56	38.95 ± 12.82	0.196
BMI (kg/m^2^)	22.97 ± 3.16	21.86 ± 3.08	0.039
Sex: male, n (%)	21 (50.0)	76 (45.5)	0.602
Chronic respiratory symptoms			
Cough, n (%)	32 (76.2)	145 (86.8)	0.087
Sputum, n (%)	18 (42.9)	70 (41.9)	0.912
Wheeze, n (%)	17 (40.5)	55 (32.9)	0.358
Dyspnea, n (%)	13 (31.0)	32 (19.2)	0.097
mMRC	0 (0, 1)	0 (0, 0)	0.493
CAT	7 (4, 9)	6 (3, 8)	0.204
Spirometry parameters			
FEV_1_ (% predicted)	95.65 ± 9.57	106.86 ± 12.04	< 0.001
FVC (% predicted)	107.09 ± 11.25	105.60 ± 13.53	0.513
FEV_1_/FVC	75.29 ± 3.10	85.92 ± 5.97	< 0.001
FEF_25–75%_ (% predicted)	57.35 ± 6.26	93.80 ± 19.18	< 0.001
FEF_50%_ (% predicted)	60.77 ± 6.11	98.58 ± 19.02	< 0.001
FEF_75%_ (% predicted)	51.62 ± 10.24	88.85 ± 19.40	< 0.001

The data are presented as mean ± SD

**Fig. 2:**
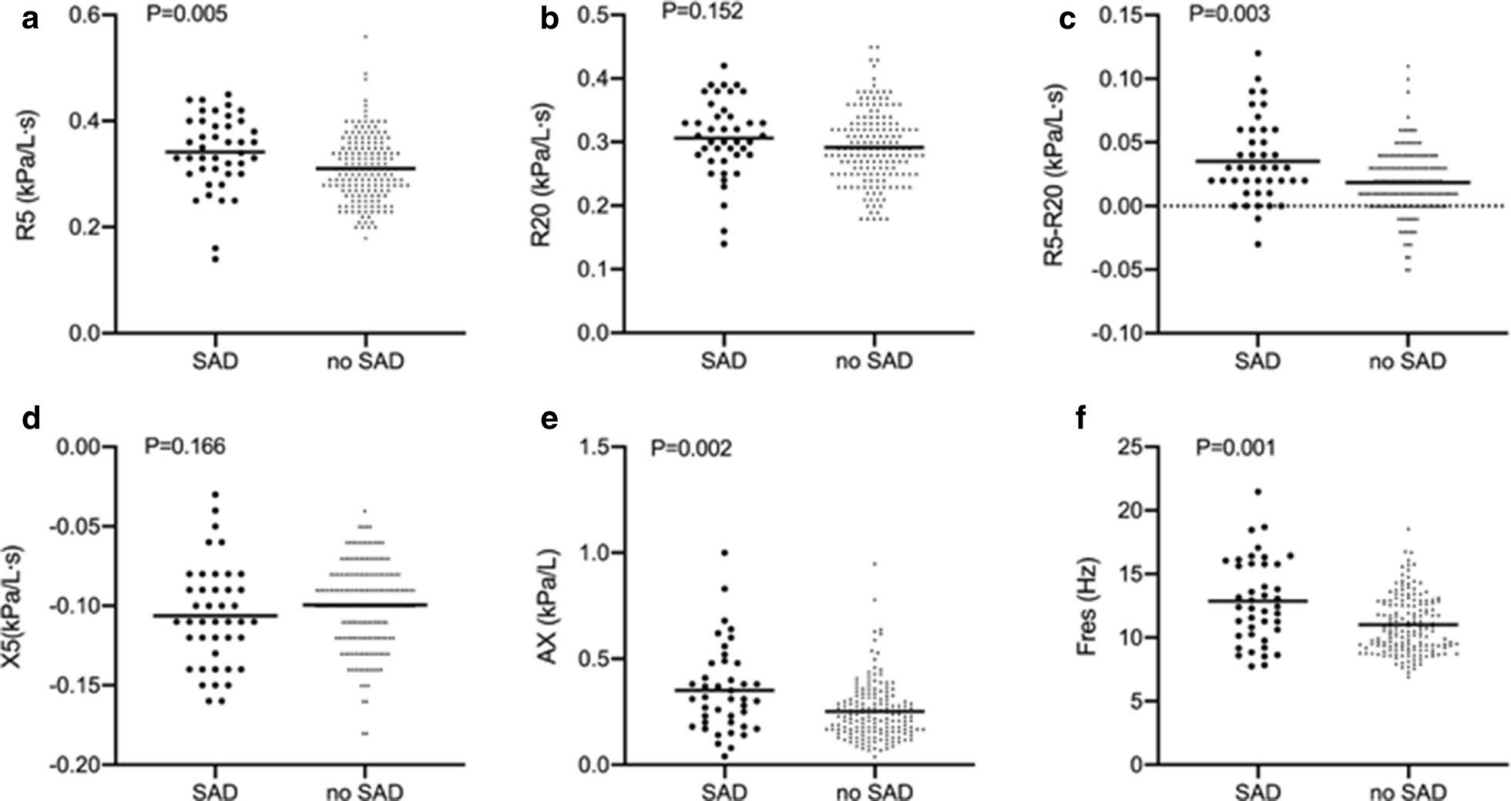
IOS parameters including **a** R5, **b** R20, **c** R5–R20, **d** X5, **e** AX and **f** Fres were plotted for subjects with chronic respiratory symptoms and PPF from the SAD versus no SAD groups. The bars represented means

IOS parameters related to small airways increased in subjects with spirometry defined SAD compared with those that didn’t have SAD (Fig. 2). R5 was a little higher in SAD group (0.34 ± 0.07 kPa/L s) than group without SAD (0.31 ± 0.06 kPa/L s, P = 0.005, Fig. 2a). R5–R20 of SAD group was nearly two times higher than that of no SAD (0.0352 ± 0.0321 kPa/L s versus 0.0185 ± 0.0250 kPa/L s, P = 0.003, Fig. 2c) while there was no difference in R20 (0.31 ± 0.06 kPa/L s versus 0.29 ± 0.06 kPa/L s, P = 0.152, Fig. 2b). Meantime, no difference in X5 was observed between two groups (− 0.1062 ± 0.0318 kPa/L s versus 0.0995 ± 0.0267 kPa/L s, P = 0.166, Fig. 2d), but AX was significantly higher in SAD subjects compared with those without it [0.31 (0.20, 0.48) kPa/L versus 0.23 (0.16, 0.31) kPa/L, P = 0.002, Fig. 2e]. Significantly increased Fres was also observed in SAD group (12.87 ± 3.30 Hz) compared with the one without SAD (11.01 ± 2.23 Hz, P = 0.001, Fig. 2f).

### The value of IOS parameters to detect SAD in the study population

**Fig. 3:**
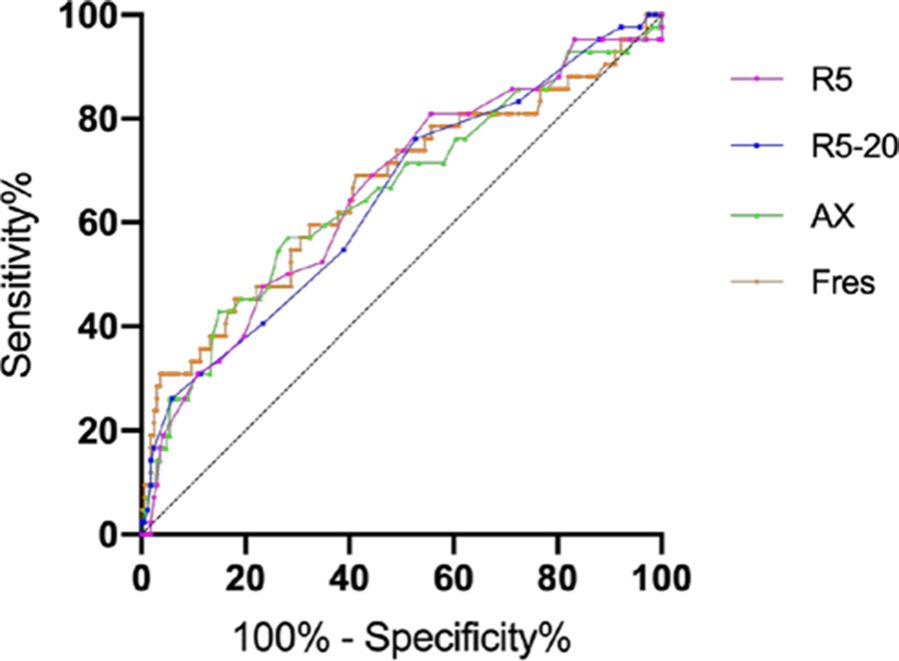
ROC curves of IOS parameters (R5, R5–R20, AX, Fres) in detecting SAD identified by small airway parameters of spirometry

With SAD standard taken as at least two of the three small airway indicators of spirometry (FEF_25–75%_, FEF_50%_ and FEF_75%_) less than 65% predicted, we conducted ROC analyses of IOS parameters which were significantly different between groups with and without SAD. And the cutoff values were determined as follows: R5 greater than 0.30 kPa/L s, R5–R20 greater than 0.015 kPa/L s, AX greater than 0.30 kPa/L, and Fres greater than 11.23 Hz (Table 5). Among these parameters, Fres had the largest AUC [0.665 (95% CI 0.564–0.766)] yield a sensitivity of 69% and specificity of 58.7%. In addition, R5 had the highest sensitivity of 81% [AUC, 0.659 (95% CI 0.563–0.754)] while AX had the highest specificity of 71.9% [AUC, 0.656 (95% CI 0.557–0.756)] (Table 5 and Fig. 3).

**Table 5:** Cutoff values of IOS parameters for the prediction of SAD in subjects with chronic respiratory symptoms and PPF (n = 209)

IOS parameter	Cutoff value	AUC	Sensitivity (%)	Specificity (%)	LR (+)	LR (−)	Youden index	P value
R5	0.30	0.659	81.0	44.3	1.45	0.43	0.25	0.002
R5–R20	0.015	0.646	76.2	47.3	1.45	0.50	0.24	0.004
AX	0.30	0.656	57.1	71.9	2.03	0.60	0.28	0.002
Fres	11.23	0.665	69.0	58.7	1.67	0.53	0.28	0.001

*AUC* area under curve, *LR* likelihood ratio

**Fig. 4:**
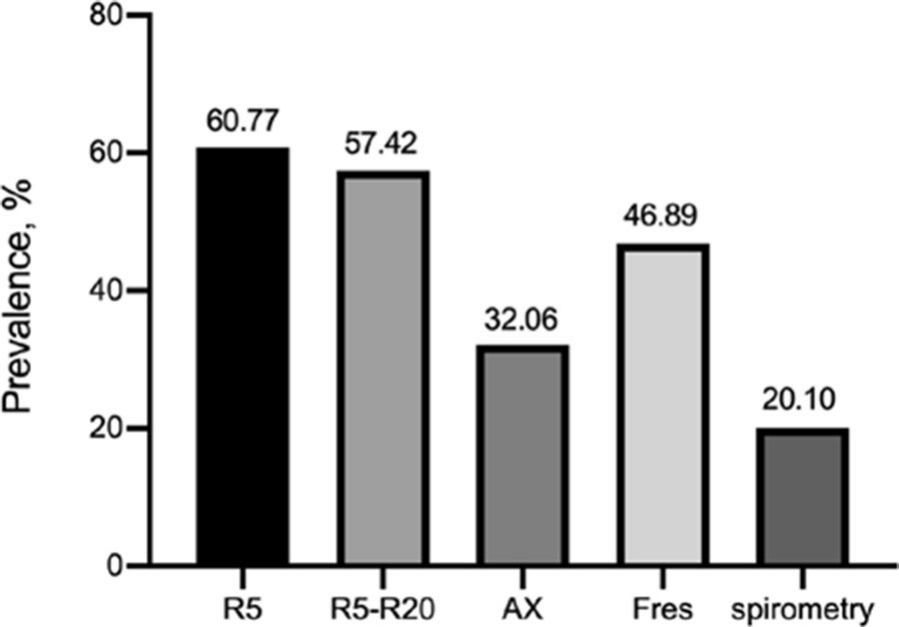
Prevalence of SAD in subjects with chronic respiratory symptoms and PPF, as measured by spirometry and different IOS parameters

Compared with spirometry, R5, R5–R20, AX and Fres could detect more SAD in subjects with chronic respiratory symptoms and PPF (Fig. 4). Among these IOS parameters, R5 determined the highest prevalence of SAD.

## Discussion

In the present study, we find that IOS might be a useful measurement for detection of SAD in subjects with chronic respiratory symptoms and PPF. Its higher sensitivity and easier implementation make up for the deficiency of spirometry. Besides, IOS parameters seem to correlate better with symptoms than spirometry.

Our target study population is symptomatic subjects with PPF. The subjects suffer from COPD/asthma-like symptoms but having normal FEV_1_/FVC which is not different from healthy controls. IOS parameters including R5, R20, Fres and AX significantly increase in symptomatic subjects. The same trend was also observed in several other studies which focus on the comparison of patients with self-reported symptoms but normal spirometry and healthy people [17], or studies comparing the symptomatic and asymptomatic PPF cohorts after environmental exposure [10, 18]. All these findings shed light on the possible location of injury which results in symptoms in subjects with PPF is more distal and could be better detected by IOS than conventional spirometry.

More and more studies [9, 11, 19] found that spirometry was not sensitive to assess small airway function. Xiao et al. [5] found that FEF_25–75%_ was often within the normal range when FEV_1_/FVC was above 75%. Besides, the forced expiration depends greatly on subject’s effort and the maneuver itself might alter bronchomotor tone [20]. In contrast, IOS could distinguish signals from different lung regions to provide comprehensive information on regional heterogeneity [11]. These might explain the reason why the correlations between spirometry and IOS parameters are not strong in the present study. Even so, IOS parameters related to small airways, namely R5, R5–R20, AX and Fres correlate relatively better with the three small airway indicators of spirometry.

When subjects were classified into with and without SAD according to the spirometry-SAD standard, exactly the above four IOS parameters associated with small airway function significantly differed between groups. Representing the total (central and distal) airway resistance, R5 may rise due to the increase of distal airway resistance [21]. R5–R20 reflects the resistance of peripheral airways and the regional ventilation heterogeneity [11]. Its value represents the degree of frequency dependency, which is usually absent in healthy adults [8]. AX is related to peripheral lung compliance and small airway patency [22]. As the frequency at which reactance is zero, meaning equal and opposite inertial and elastic properties at this frequency [23], Fres reflects the capacitance and inertial properties of peripheral airways [11]. Except for the above four parameters, decreases in X5 were also observed in other studies [17, 19, 24] which compared subjects with and without symptoms or SAD. However, X5 is not different between SAD and no SAD group in the present study. Reflecting the dynamic compliance of respiratory system, it is affected not only by the elasticity of respiratory system itself but also by the airflow resistance i.e. time constants [8, 25]. The failure to find a difference in X5 may be due to the unimpaired elasticity and lung volume. Besides, the relatively mild symptoms and lesions in our study population might represent that the degree of expiratory flow limitation (EFL) is relatively mild. However, EFL manifests in the difference of reactance between inspiratory and expiratory phase, while X5 reflects the mean reactance during the whole breath.

Compared with spirometry, the prevalence of SAD is higher when it is recalculated with IOS cutoff values, which has also been confirmed in previous studies [10, 11, 19, 24]. IOS could detect airway dysfunction that spirometry failed to [9, 26]. Consistent with the prior study [24], Fres seems to perform the best in diagnosing SAD on account of the largest AUC. However, R5 and R5–R20 have the highest sensitivity and might be more suitable as screening indexes. When it comes to the exact diagnosis in clinic, either high sensitivity or specificity of the individual index might demand a comprehensive consideration of various IOS and spirometry indexes. In addition, the cutoff values obtained in our study are generally lower than those in previous studies. For R5–R20, abnormal cutoff values were concluded as 0.03 kPa/L s [26] or 0.07 kPa/L s [24, 27], greatly different from the 0.015 kPa/L s in the present study. A study [28] used the upper limit (0.35 kPa/L s) of above 400 normal subjects as the critical value for R5, which was also a little higher than our value of 0.30 kPa/L s. The interindividual variation of IOS values, which is not good as spirometry in this regard [12], together with the different SAD standards, disease severity, and race of the study population might result in the differences of cutoff values.

Apart from the high sensitivity, IOS also performs better in the association with specified symptoms than spirometry. R5–R20 and Fres are significantly increased in subjects with sputum than those without. Similarly, subjects with dyspnea have higher R5, R5–R20 and AX. The above parameters are all related to peripheral airways. Sputum production reflects the increase of inflammatory mediators in the lung. Previous results were consistent with our findings, suggesting that the place of airway inflammation, obstruction and injury in such symptomatic patients with PPF is predominated with small airways [17, 24]. Except for the increased FEF_75%_ in subjects with dyspnea, most spirometry parameters failed to identify the difference between subjects with and without specified symptoms. We also evaluated the symptoms quantitatively using mMRC and CAT scores to explore the correlation of IOS and spirometry results with the severity of symptoms. Although the relationships between severity of dyspnea and IOS parameters are weak and fail to survive Bonferroni correction, no relationship is observed for spirometry results. Besides, Bonferroni correction is so strict that could result in the possibility of false negativity. The correlations between comprehensive evaluation for all symptoms (CAT score) and IOS parameters are also stronger than spirometry indicators. In spite of previous use of CAT score in ever-smokers with PPF in the literature [29], it seems not appropriate to the present study population with relatively mild symptoms. As the symptom score applying to COPD patients, it may be more suitable for patients with more obvious symptoms and a higher risk of poor outcomes. Studies with large sample sizes are needed to identify IOS indicators related to symptoms. IOS reflects the pulmonary structure and mechanical characteristics during normal breathing rather than the effort-dependent forced expiratory, which might be the explanation of the better correlation for IOS with symptoms.

Overall, despite the controversial evidence on whether SAD could predict the development of COPD or asthma, it is of great importance to make a comprehensive evaluation of small airways in the early disease stage. Early detection facilitates early intervention such as smoking cessation and the utilization of small particle aerosols targeting peripheral lung. Meantime, sensitivity to changes in individuals and easy execution make IOS more suitable for long-term monitoring.

Our study has several other limitations except for the limited sample. First, we used spirometry defined SAD as the reference criterion to explore the diagnostic value of IOS. Currently, no consensus has been reached about the gold standard for SAD diagnosis. Some more intuitive detection method such as endobronchial optical coherence tomography (EB-OCT) might be more objective as reference. Second, having no predicted values adjusted by age, sex and BMI, IOS results are absolute values of individuals, which probably affects the comparability between groups. In addition, although objective languages were used in the questionnaire, the evaluation of symptoms might still be subjective. Finally, we didn’t track the subjects’ disease progression and treatment responses in spirometry and IOS parameters. We still don’t know whether these detected SADs would develop into exact diseases like COPD or asthma. Prospective studies with a large sample size of population and more suitable scores for symptoms are needed to further explore the application of IOS in diagnosing and monitoring SAD.

## Conclusion

To summarize, the current study shows that IOS is more sensitive to detect SAD than spirometry in subjects with chronic respiratory symptoms and PPF. Our data also find a better relationship for IOS parameters with symptoms in these subjects. Moreover, IOS is easy to perform and provides extended information on the central and peripheral lung. Hence, IOS could be an additional method for SAD detection in the early stage of diseases.

## Data Availability

All the data will be available to other researchers on reasonable requests to the corresponding author after publication.

## Abbreviations

COPD: Chronic obstructive pulmonary disease
SAD: Small airway dysfunction
PPF: Preserved pulmonary function
FEV_1_: The forced expiratory volume in 1st s
FVC: Forced vital capacity
AHR: Airway hyper-responsiveness
BR: Bronchial reversibility
FEF_25–75%_: The forced expiratory flow between 25 and 75% of FVC
FEF_50%_: The forced expiratory flow when 50% of FVC has been exhaled
FEF_75%_: The forced expiratory flow when 75% of FVC has been exhaled
IOS: Impulse oscillometry
Rrs: Resistance of the respiratory system
Xrs: Reactance of the respiratory system
R5: Respiratory resistance at 5 Hz
R20: Respiratory resistance at 20 Hz
R5–R20: The difference between R5 and R20
X5: Reactance at 5 Hz
Fres: Resonant frequency
AX: Area under reactance curve between Fres and 5 Hz
ATS: American Thoracic Society
ERS: Europe Respiratory Society
PD_20_FEV_1_: Provocative dose inducing a 20% decrease of FEV_1_
mMRC: Modified Medical Research Council dyspnea scale
CAT: COPD Assessment Test
ROC: Receiver operator characteristic
AUC: Area under the curve
BMI: Body mass index
LR: Likelihood ratio
EFL: Expiratory flow limitation
EB-OCT: Endobronchial optical coherence tomography
